# Detection of acute toxicity of aflatoxin B1 to human hepatocytes *in vitro* and *in vivo* using chimeric mice with humanized livers

**DOI:** 10.1371/journal.pone.0239540

**Published:** 2020-09-23

**Authors:** Yuji Ishida, Chihiro Yamasaki, Hiroko Iwanari, Hisahiko Yamashita, Yuko Ogawa, Ami Yanagi, Suzue Furukawa, Yuha Kojima, Kazuaki Chayama, Junichi Kamiie, Chise Tateno

**Affiliations:** 1 Department of Research and Development, PhoenixBio Co., Ltd., Higashi-Hiroshima, Hiroshima, Japan; 2 Research Center for Hepatology and Gastroenterology, Hiroshima University, Hiroshima, Hiroshima, Japan; 3 Quantitative Biology and Medicine, Research Center for Advanced Science and Technology (RCAST), The University of Tokyo, Meguro, Tokyo, Japan; 4 Institute of Immunology Co., Ltd., Bunkyo, Tokyo, Japan; 5 Department of Gastroenterology and Metabolism, Applied Life Sciences, Institute of Biomedical and Health Sciences, Hiroshima University, Hiroshima, Hiroshima, Japan; 6 Laboratory of Veterinary Pathology, School of Veterinary Medicine, Azabu University, Sagamihara, Kanagawa, Japan; University of Navarra School of Medicine and Center for Applied Medical Research (CIMA), SPAIN

## Abstract

Aflatoxin B1 (AFB1), a mycotoxin, is acutely hepatotoxic to many animals including humans. However, there are marked interspecies differences in sensitivity to AFB1-induced toxicity depending on bioactivation by cytochrome P450s (CYPs). In the present study, we examined the applicability of chimeric mice with humanized livers and derived fresh human hepatocytes for *in vivo* and *vitro* studies on AFB1 cytotoxicity to human hepatocytes. Chimeric mice with highly humanized livers and SCID mice received daily injections of vehicle (corn oil), AFB1 (3 mg/kg), and carbon tetrachloride (50 mg/kg) for 2 days. Histological analysis revealed that AFB1 promoted hepatocyte vacuolation and inflammatory cell infiltration in the area containing human hepatocytes. A novel human alanine aminotransferase 1 specific enzyme-linked immunosorbent assay demonstrated the acute toxicity of AFB1 to human hepatocytes in the chimeric mouse livers. The sensitivity of cultured fresh human hepatocytes isolated from the humanized liver mice for AFB1 cytotoxicity was comparable to that of primary human hepatocytes. Long-term exposure to AFB1 (6 or 14 days) produced a more severe cytotoxicity. The half-maximal lethal concentration was 10 times lower in the 2-week treatment than after 2 days of exposure. Lastly, the significant reduction of AFB1 cytotoxicity by a pan-CYP inhibitor or transfection with CYP3A4 specific siRNA clearly suggested that bioactivation of AFB1 catalyzed by CYPs was essential for AFB1 cytotoxicity to the human hepatocytes in our mouse model. Collectively, our results implicate the humanized liver mice and derived fresh human hepatocytes are useful models for studies of AFB1 cytotoxicity to human hepatocytes.

## Introduction

Aflatoxins are a group of secondary metabolites produced by *Aspergillus* fungi, such as *A*. *flavus* and *A*. *parasiticus*. Although the exact function of the aflatoxins have not been cleared, production of the aflatoxins is regulated by at least 30 genes and significantly affected by several environmental factors (such as light, pH and carbon sources), it is postulated that production of aflatoxins provides fungi with some benefits in growth [1]. The aflatoxins are also toxic compounds classified by the International Agency for Research on Cancer in the highest risk group with sufficient evidence of carcinogenicity in humans [2–4]. Among about 20 known aflatoxins, aflatoxin B1 (AFB1) is considered as one of the most potent inducers of acute and chronic liver injury and hepatocellular carcinoma [5]. In the past several decades, the consumption of AFB1-contaminated food (such as corn, peanuts, milo, sorghum, copra, and rice) has caused many episodes of AFB1-related human deaths [2]. This has spurred many prior and ongoing studies to develop effective intervention methodologies to mitigate the health impacts of AFB1 exposure, including the development of biomarkers correlated with exposure level, establishment of sensitive detection methods, and protective interventions against exposure [2].

The mode of action of AFB1-induced liver injury has been described [2, 3]. AFB1 is considered a pro-toxic chemical. One of its metabolites, AFB1-8,9-epoxide (AFBO), is essential for carcinogenic and hepatotoxic activity [3]. This bioactivation of AFB1 is catalyzed by cytochrome P450s (CYPs), and the reactive metabolite is finally detoxicated by glutathione conjugation catalyzed by glutathione-S-transferases (GSTs) [3]. The liver toxicity of AFB1 is modulated by a balance of formation and detoxication of the reactive metabolites in hepatocytes [6]. The diverse liver functions among different animals are reflected in the significant differences in severity of AFB1-induced acute liver injury among them [2, 3]. The marked difference is evident even in rodents. Mice display extremely low sensitivity to liver toxicity of AFB1 due to high metabolic activity of GSTA3 toward AFB [7], whereas rats are among the animals that are most sensitive to AFB1-induced liver toxicity and carcinogenicity [8, 9]. Such interspecies differences in the susceptibility to AFB1-induced liver toxicity restrict the use of experimental animal models for human related studies. In contrast, human hepatic cell lines are considered more reliable models for studies of AFB1 cytotoxicity reflecting human drug metabolism. However, there are significant differences in hepatocyte functions, including drug metabolism capacities, among these cell types and primary human hepatocytes (HHs) [10–12]. Although primary HHs are well-accepted tools for studies of AFB1 liver toxicity in humans, there are many limitations to the use of primary HHs, such as restricted supply volume and lot-to-lot variations [12, 13].

Humanized liver chimeric mice have been produced by transplantation of normal HHs into genetically engineered immunodeficient/liver injured host mice [14]. We reported that chimeric mice generated from albumin enhancer/promoter-driven cDNA urokinase-type plasminogen activator/severe combined immunodeficiency (cDNA-uPA/SCID) mice (PXB-mice) exhibited stable repopulation rates of HHs for several months [15], and were useful for studies on drug metabolism and pharmacokinetics, drug-drug interactions [16–18], and chronic infection by hepatitis viruses [19]. PXB-mice and the other type of mice with humanized livers have been utilized for *in vivo* toxicology studies, with human specific cytotoxicity reported for troglitazone, fialuridine, and bosentan [20–23]. Moreover, we reported that fresh HHs isolated from the PXB-mice (PXB-cells) could be readily plated and cultured for several weeks in a conventional culture condition [24]. PXB-cells cultured at high cell density displayed pronounced elevations in activities of CYPs and transporters, metabolic enzyme induction, and stable susceptibility to hepatitis B virus infection *in vitro* [24–27].

Based on these lines of evidence, we hypothesized that the PXB-mice and derived PXB-cells would be suitable *in vivo* and *in vitro* models, respectively, for the analysis of AFB1 liver toxicity in humans. To test this hypothesis, we evaluated the *in vivo* acute toxicity of AFB1 to HHs in livers of PXB-mice by histological examination and a novel enzyme-linked immunosorbent assay (ELISA) specific for human alanine transferase 1 (hALT1). Moreover, the dose- and time-dependent AFB1 cytotoxicity to PXB-cells were assessed *in vitro* by analyzing the cell viability with real-time cell analysis (RTCA) and WST-1 assays. Finally, we analyzed the effects of a non-specific CYP inhibitor and transfection of CYP3A4 small interfering RNA (siRNA) on AFB1-induced cytotoxicity to PXB-cells. Our results demonstrate that the humanized liver chimeric mice and derived HHs are a reliable *in vivo* and *in vitro* research tool for studies of AFB1 toxicity for HHs.

## Materials and methods

### Ethics statements

Human hepatocytes were purchased from BD Biosciences. The cells were obtained with written informed consent. This research was approved by Utilization of Human Tissue Ethical Committee of PhoenixBio (0028). All experimental procedures were conducted in accordance with the guidelines provided by the Proper Conduct of Animal Experiments (June 1, 2006; Science Council of Japan) and were approved by the Animal Care and use Committee of the Institution (1205). All surgical procedures (i.e., cell transplantation), blood sampling, and necropsy were conducted under isoflurane (Escain®, Mylan Inc.) anesthesia. For *in vivo* study, mice were sacrificed by cardiac puncture and exsanguination under anesthesia at the endpoint. All experimental animals were housed with environmental enrichments under pathogen-free conditions and maintained in a 12-h light/dark cycle with sterilized water and regular diet CRF-1 (Oriental Yeast Co., Ltd.) available *ad libitum*.

### Chemicals and animals

Corn oil, AFB1, carbon tetrachloride (CCl_4_), and aminobenzotriazole (ABT) were purchased from Wako Pure Chemical Industries Ltd, LKT Laboratories, Inc., and Sigma-Aldrich, respectively.

**Fig 1:**
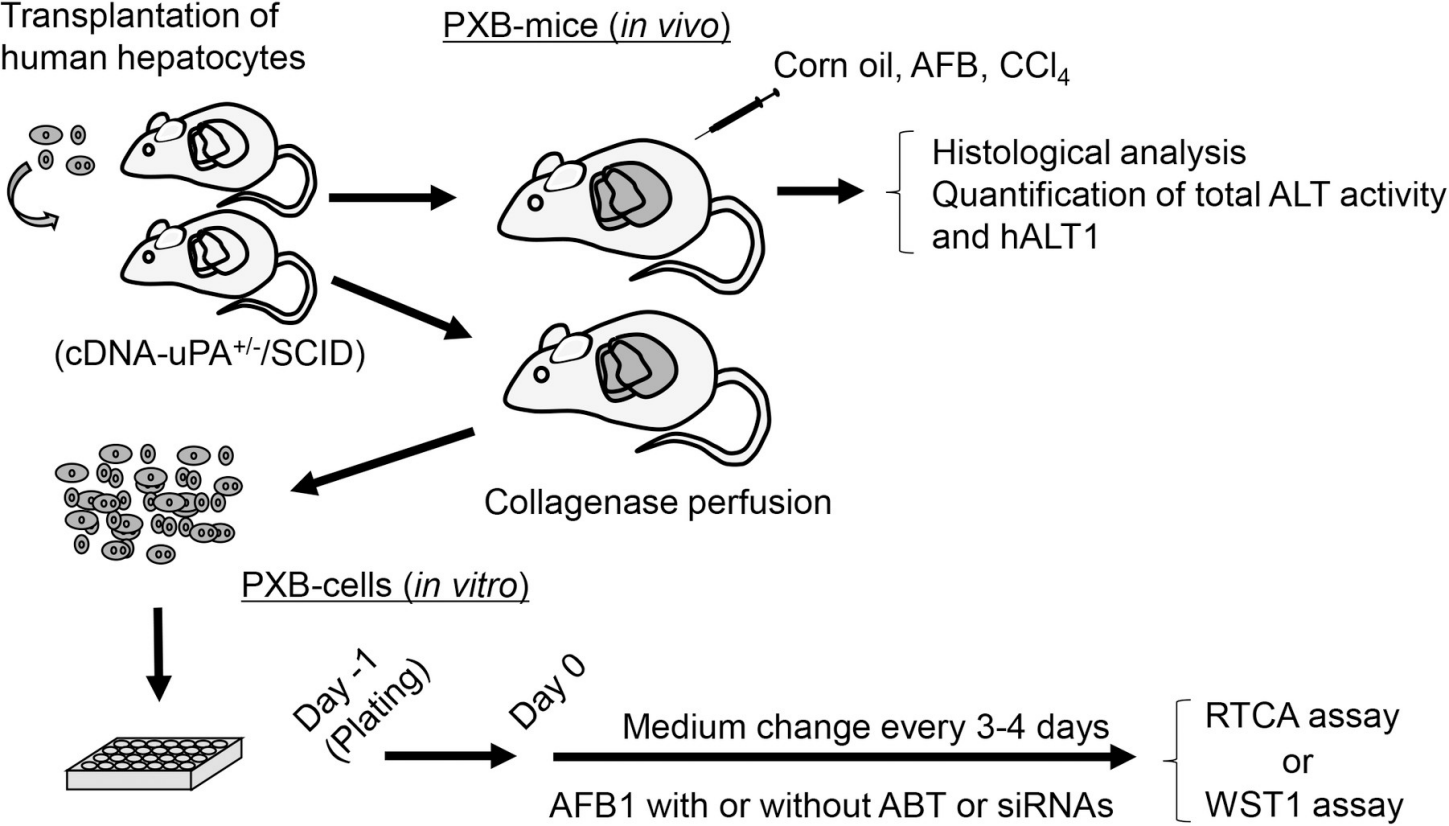
Summary of *in vivo* and *in vitro* experiments for AFB1-induced human hepatotoxicity with PXB-mice and PXB-cells.

SCID (C.B-17) mice were purchased from CLRA Japan, Inc. A total of 13 SCID mice were used for *in vivo* study. For construction of the chimeric mice, transplantation of the cryopreserved HHs (Lot: BD195, 2-year-old Hispanic girl; BD Bioscience) into the cDNA-uPA^+/-^/SCID mice, monitoring of blood human albumin levels, and estimation of replacement index (RI) of HHs in mouse liver were performed as previously described [15]. We used humanized liver mice (13 mice for *in vivo* studies, 28 mice for *in vitro* studies) highly repopulated (RI >70%) with the transplanted HHs (Fig 1).

PXB-mice were produced by xenotransplantation of human hepatocytes into recipient mice (cDNA-uPA^+/-^/SCID mice) followed by chemical administration or collagenase perfusion. For *in vitro* experiments, PXB-cells were isolated from the PXB-mice transplanted with the same donor hepatocytes and treated with AFB1 with or without a CYP inhibitor co-treatment or CYP3A4 specific siRNA transfection.

### Establishment of sandwich ELISA with anti-hALT1 mAbs

Materials and methods for preparation of ALT samples and establishment of mouse anti-human ALT1 monoclonal antibodies (anti-hALT1 mAbs) were described in S1 File. Optimal buffer, time, and temperature incubation conditions, as well as the optimal concentrations of antibodies were determined. The finalized hALT1 sandwich ELISA was developed as described next.

Anti-hALT1 mAb TA2154-100 was used to coat the wells of a 96-well microtiter plate Maxisorp (Thermo Fisher Scientific) and allowed to adhere overnight at 4°C and blocked with the assay buffer of phosphate buffered saline (PBS) containing 1% bovine serum albumin at room temperature (RT) for 1 h. Each well was washed five times with PBS-containing 0.05% Tween-20 (PBS-T). Dilutions (1:10 in assay buffer) of purified recombinant hALT1 and hALT2 (rhALT1, rhALT2 respectively), or human serum and serum collected from SCID mice received hydrodynamic injection (HDI-SCID serum), or the standard of hALT1, which were serially diluted in normal mouse serum (Equitech-Bio, Inc.), or individual human or mouse serum samples were added to the wells. Plates were incubated by shaking at 700 rpm for 1 h at 25°C. After three washes with PBS-T, biotin conjugated anti-hALT1 mAb TA2214-116 (Biotin-TA2214-116) was added to each well and incubated as before. Biotin-TA2214-116 was prepared using the EZ-Link^TM^ Sulfo-NHS-LC-Biotinylation kit (Thermo Fisher Scientific) according to the manufacturer’s instructions. After three washes with PBS-T, avidin D conjugated-horseradish peroxidase (Avidin D-HRP, Vector Laboratories) was added to each well and incubated as before. Each well was washed five times with PBS-T, and BioFX TMB colorimetric substrate (SurModics) was added and incubated at 25°C for 30 min in the dark. The reaction was terminated by the addition of BioFX 450 nm liquid stop solution (SurModics). The optical density in each well was determined at 450 nm (A450) using a SpectraMax 340P device (Molecular Devices). A calibration curve was plotted and the concentration of hALT1 were estimated by the least squares method.

### Animal study and histological analysis

Male PXB-mice and SCID mice were randomly selected to receive corn oil (vehicle, n = 5), AFB1 (n = 5), or CCl_4_ (n = 3). AFB1 and CCl_4_ were suspended in corn oil. Each mouse received an intraperitoneal injection of corn oil (5 mL/kg), AFB1 (3 mg/5 mL/kg), or CCl_4_ (50 mg/5 mL/kg) once daily for 2 days. Each injected dose was determined based on previous studies [28, 29]. Blood samples (50 μL) were collected from each animal under isoflurane anesthesia at day 0 (before dosing) and day 2. Collected blood samples were kept frozen at -80°C until analysis. All mice were necropsied and liver tissues were collected at day 2. Liver slices approximately 5 mm thick were prepared and immersed overnight in 10% formalin neutral buffer solution. Formalin fixed liver tissues were embedded in paraffin and sectioned at a thickness of 5 μm for immunohistochemistry and hematoxylin and eosin staining. For the immunostaining of Gr-1 (also termed Ly6-G/C), liver sections were processed for antigen retrieval (Target Retrieval solution, Dako) for 20 min and incubated with primary antibody (#550291, BD Pharmingen) overnight at 4°C. Secondary antibody (Histofine simple stain mouse MAX-PO (RAT), Nichirei Bioscience) was applied for 30 min at RT. Finally, nuclei were counterstained with hematoxylin and slides were observed using an Eclipse E800 microscope (Nikon).

### Culture and viability analysis of PXB-cells

PXB-cells were isolated from PXB-mice transplanted with the same donor hepatocytes used for the *in vivo* study and cultured as previously reported [24, 27] (Fig 1). PXB-cells were plated (2.1 × 10^5^ cells/cm^2^) on type I collagen-coated 24-well plates (BioCoat, Corning) or E-plate L8 (ACEA Biosciences). One day after plating (Day 0), the seeding medium (Dulbecco’s modified Eagle’s medium containing 10% of fetal bovine serum) was changed to a maintenance medium (hHCGM [24]), containing AFB1 at the indicated concentrations in the presence or absence of ABT (125 or 500 μM), a non-specific CYP inhibitor. The medium was changed twice a week. Cell viability was analyzed using the RTCA iCELLigence^TM^ (ACEA Biosciences) or WST-1 assay (Cell proliferation reagent WST-1, Roche) according to the manufactures’ instructions.

### Transfection of siRNAs

Negative control and CYP3A4 specific siRNAs were purchased from Dharmacon (siGENOME SMARTpool). Transfection of siRNAs was conducted by seeding PXB-cells on wells coated with these siRNAs (prepared by CytoPathfinder) as previously described [24]. PXB-cells were treated with AFB1 as indicated dose for 6 days beginning 8 days after transfection.

### Measurement of CYP 3A activity

CYP3A activity was measured using midazolam, a specific substrate for CYP3A, using high-performance liquid chromatography (HPLC, Lachome Elite; Hitachi High-Technology Co.) as previously described [30]. Briefly, after washing with PBS, cells were cultured with Krebs-Henseleit buffer containing 10 mM midazolam for 2 h. After incubation, the supernatant was collected and the concentration of 1’-hydroxymidazolam was measured using HPLC. HPLC was performed at a flow rate of 1.0 mL/min using an Xterra RP18 column (4.6´150 mm, 5 μm; Waters).

### Real-time quantitative PCR (RT-qPCR)

RNA was extracted with Trizol (Thermo Fisher Scientific) reagent according to the manufacture’s instruction. One microgram total RNA was used as template for reverse transcription with SuperScript^TM^ III First-Strand Synthesis System (Thermo Fisher Scientific). RT-qPCR was conducted using the SYBR Green Master Mix (Applied Biosystems) and human CYP3A4 specific primers as previously described [31, 32].

### Statistical analysis

Statistical analysis was performed using Statcel 4 (OMS Publishing Inc.). Results are expressed as mean ± SD, and the significance of the difference between two groups was analyzed using the Student’s t-test when data were normally distributed, else using the Welch’s t-test. Multiple-sample comparisons were made using one-way analysis of variance followed by post hoc analysis. *p*-values <0.05 were considered statistically significant.

## Results

### Detection of human specific AFB1-induced hepatotoxicity *in vivo*

**Fig 2:**
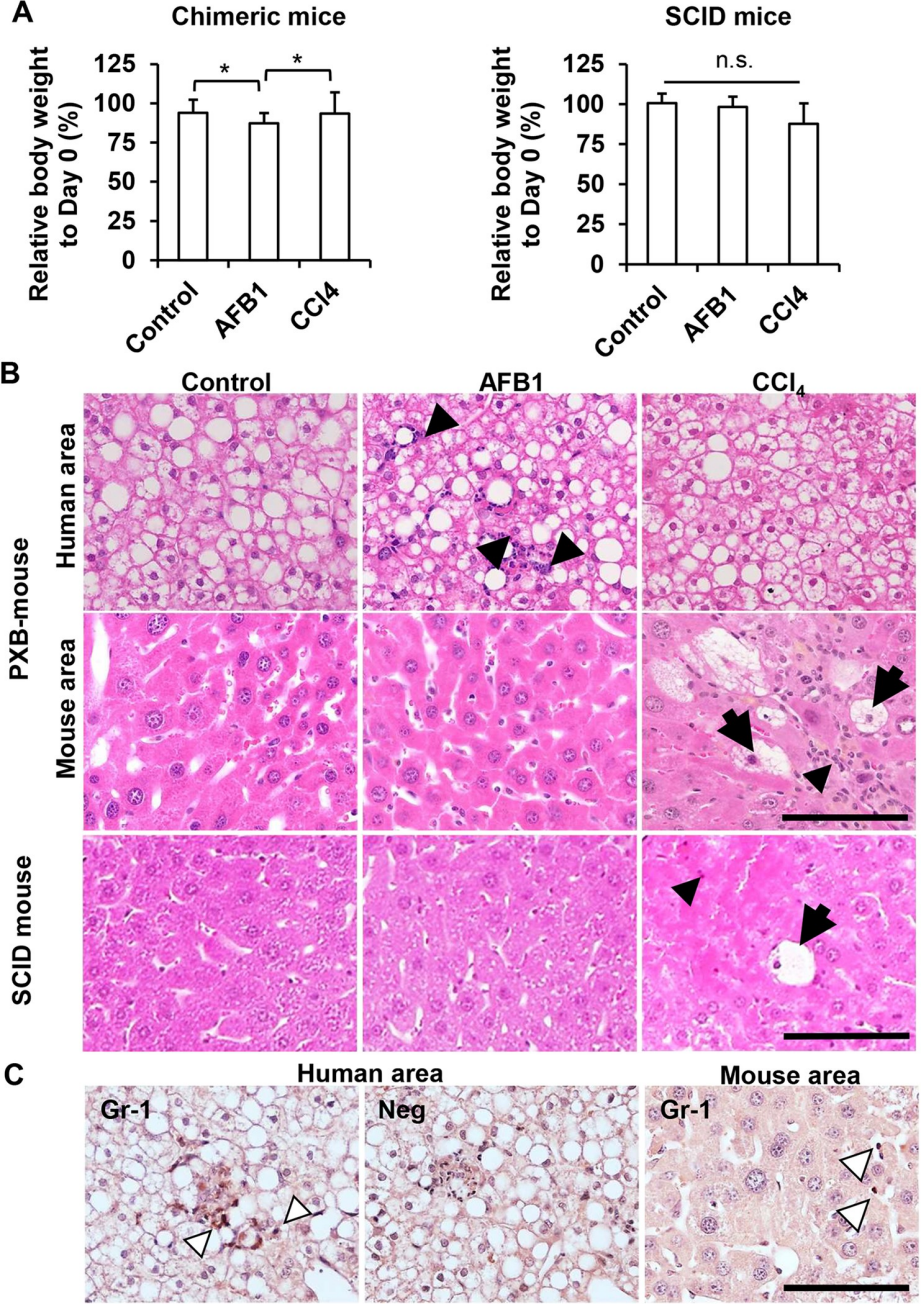
AFB1-induced human hepatotoxicity in PXB-mice. Male PXB-mice and SCID mice received daily intraperitoneal administration of corn oil (n = 5), AFB1 (n = 5), or CCl_4_ (n = 3) for 2 days. All animals were necropsied 24 h after the final injection. (A) Relative body weight to initial value. Values are means ± S.D. *p <0.01 (Tukey-Kramer test). n.s., not significant (*p* >0.05, Steel-Dwass test). (B) Liver sections stained with hematoxylin and eosin. Each animal was treated with the indicated chemical. Upper, middle, and lower panel represents human and mouse hepatocyte area in the PXB-mice, and in the SCID mouse livers, respectively. Arrows and arrowheads represent vacuolated hepatocytes and infiltrated inflammatory cells, respectively. Scale bars, 100 μm. (C) Representative images of Gr-1 stained liver sections collected from PXB-mice injected with AFB1. White arrowheads indicate Gr-1-positive cells. Neg; Negative control. Scale bar, 100 μm.

To examine the cytotoxic effect of AFB1 on HHs in chimeric mouse livers, PXB-mice and SCID mice received an intraperitoneal injection of vehicle (corn oil), AFB1, or CCl_4_. The latter has also been demonstrated to produce species-dependent severe hepatotoxicity [33]. Detailed information of the PXB-mice utilized for the *in vivo* study are provided in Table 1. After daily administration of these chemicals for 2 days, all animals were necropsied and liver tissues and blood were analyzed. Except for a slight body weight reduction in AFB1-treated PXB-mice, there was no significant change in body weight in each group (Fig 2A). Histological examination clearly demonstrated that administration of AFB1 promoted hepatocyte vacuolation and infiltration of Gr-1-positive inflammatory cells in the area containing HHs, but not in the remaining host mouse hepatocyte area (Fig 2B and 2C). On the other hand, hepatocyte cell death and inflammatory cell infiltration were specifically observed in mouse hepatocyte area in CCl_4_-injected livers of PXB-mice (Fig 2B). Consistent with these results, CCl_4_, but not AFB1, induced hepatocyte cell death and inflammatory cell infiltration in livers of the SCID mice.

**Table 1:** Animal information.

Group	ID	Age (weeks)	Blood hAlb (mg/mL)	RI (%)	Body weight (g)
Corn oil	1	15	12.8	92.6	18.1
	2	16	12.7	92.4	19.2
	3	15	14.9	97.2	19.1
	4	16	11.5	89.4	22.0
	5	15	15.1	97.6	18.2
AFB1	6	15	12.5	91.9	20.2
	7	15	12.5	91.9	18.1
	8	14	12.4	91.7	19.9
	9	15	13.0	93.1	20.9
	10	14	15.0	97.4	19.6
CCl_4_	11	15	7.4	76.5	23.5
	12	15	7.4	76.5	23.6
	13	15	10.2	85.9	17.9

**Fig 3:**
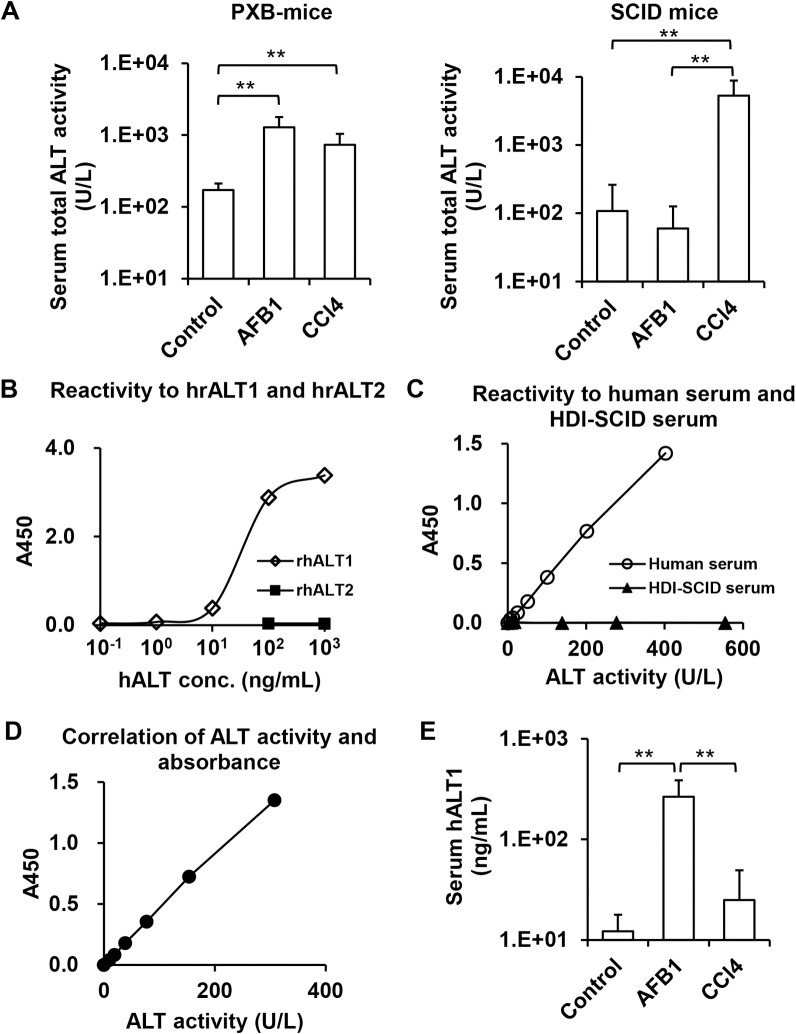
Quantification of plasma total ALT activity and hALT1 levels. (A) Plasma total ALT activity levels in PXB-mice and SCID mice were measured with a conventional method detecting both human- and mouse-derived ALTs. (B) Reactivity of the presently developed hALT1-specific ELISA to rhALT1 and rhALT2. Serially diluted rhALT1 and rhALT2 were measured using the hALT1-specific ELISA. (C) Reactivity of the hALT1-specific ELISA to samples derived from human or mouse. Levels of hALT1 and total ALT activity in serially diluted human patient sera with liver disease and mouse sera collected from HDI-SCID mice were measured with the conventional method (x-axis) and the presently developed hALT1-specific ELISA (y-axis), respectively. (D) Correlation of serum ALT activity and hALT1 levels. Total ALT activity and hALT1 levels in serially diluted gold standard serum were measured with the conventional method (x-axis) and the present hALT1-specific ELISA (y-axis). (E) Serum hALT1 levels in the PXB-mice were measured with the hALT1-specific ELISA. Values are means ± S.D. **; *p* <0.01 (Tukey-Kramer test).

Hepatotoxicity of the tested chemicals were also evaluated with plasma ALT levels, a well-accepted biomarker correlated with degree of hepatocyte injury. First, we measured the plasma total ALT activity with a conventional assay that can detect both human- and mouse-derived ALT activities [21]. Reflecting the histological changes in livers treated with the chemicals, significant increase of serum ALT activity was confirmed in PXB-mice treated with AFB1 or CCl_4_ and SCID mice treated with CCl_4_, but not AFB1-treated SCID mice (Fig 3A). These results suggested that AFB1 administration specifically damaged HHs in the humanized liver, and leakage of ALTs from the injured HHs caused a significant increase of serum ALT activity, while CCl_4_ showed mouse hepatocyte specific cytotoxicity.

To confirm the human specific hepatotoxicity of AFB1 using a quantitative biochemical assay, we tried to establish a sandwich ELISA using two different mAbs specific for hALT1. Fifteen hybridoma cell lines producing anti-hALT1 mAbs were generated. Their binding activity and specificity to rhALT1 and rhALT2 were tested at antibody concentrations ranging from 1 ng/mL to 3 μg/mL using direct ELISA. Each antibody reacted with rhALT1 in a concentration-dependent manner, but did not react with rhALT2. The specificity was confirmed by an immunoblot against rhALT1, rhALT2, and mALTs (CCl_4_-SCID). Each mAb also specifically recognized rhALT1, but did not react with rhALT2 and mALTs. We used two mAbs (TA2154-100 and TA2214-116) subclassified as IgM(κ), IgA(κ), respectively.

The specificity of the novel hALT1 sandwich ELISA was tested using purified rhALT1 and rhALT2 serially diluted in the concentration range of 0.1 to 1000 ng/mL and 100 to 1000 ng/mL respectively. The reaction of rhALT-1 displayed a good sigmoid dose-response curve, but rhALT2 unreactive (Fig 3B). Next, the specificity against hALTs was tested in comparison with mALTs. The serially diluted human serum and HDI-SCID serum prepared at ALT activities ranging from 6 to 402 U/L and 9 to 554 U/L, respectively, were applied to the hALT1 sandwich ELISA system. The human serum displayed a good dose-response curve in the tested range. Conversely, HDI-SCID serum unreactive (Fig 3C). Finally, the serially diluted hALT1 gold standard was prepared at concentrations ranging from 10 to 615 U/L in normal mouse serum. When applied to the hALT1 sandwich ELISA system, a good dose-response was evident in the range of 10 to 615 U/L (Fig 3D), allowing the establishment of a calibration curve in this ELISA system.

Using this hALT1 specific sandwich ELISA system, we measured serum hALT1 levels in PXB-mice treated with vehicle or AFB1 or CCl_4_. No significant difference in the serum hALT1 levels was observed between control (9.1 ± 4.2 ng/mL) and CCl_4_ (25.0 ± 24.3 ng/mL) treated mice. However, AFB1-treated PXB-mice showed significantly higher serum hALT1 levels (197.9 ± 90.2 ng/mL) than control and CCl4 treated mice (Fig 3E). The histochemical and biochemical analyses revealed an obvious cytotoxicity of AFB1 to HHs, but not to mouse hepatocytes, in humanized liver chimeric mice.

### *In vitro* assay of AFB1 cytotoxicity to PXB-cells

**Fig 4:**
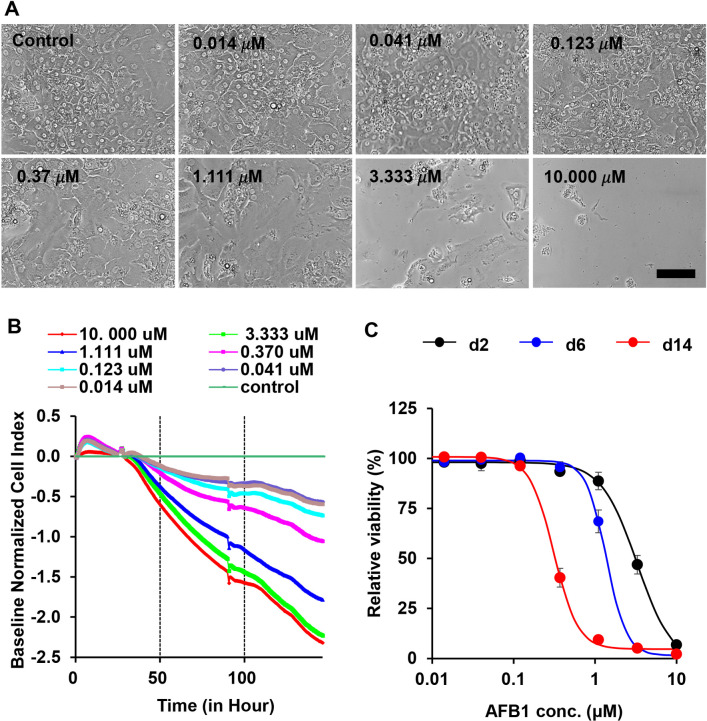
Dose-dependent cytotoxicity of AFB1 on PXB-cells. (A) Representative images of PXB-cells treated with AFB1 indicated concentrations for 5 days. Scale bar; 100 μm. (B) Cytotoxicity curves of PXB-cells exposed to AFB1 at different doses for 150 h. Cell index curves were generated with the iCELLigence software. (C) Dose-response curves of PXB-cells exposed to AFB1 for 2, 6, and 14 days. Cell viabilities were measured with the WST-1 assay.

Cytotoxicity of AFB1 to HHs was also evaluated with an *in vitro* model using PXB-cells comprising fresh HHs isolated from PXB-mice. As shown in Fig 1, AFB1 treatment was started one day after plating and extended until the end of culture. In the absence of AFB1 treatment, there was no obvious change in number of cultured PXB-cells. They maintained a mature hepatocyte morphology at least until day 14 (Fig 4A), as previously reported [24]. However, AFB1 treatment significantly reduced the number of cultured PXB-cells in a dose-dependent manner (Fig 4A, day 5). The dose-dependent cytotoxicity of AFB1 to HHs was also confirmed using the RTCA assay (Fig 4B). The half-maximal lethal concentration (LC_50_) value for PXB-cells at day 2 (2.09 ± 0.40 μM) was significantly lower than the LC_50_ for HepG2 and HepaRG cells, and comparable to that of primary HHs reported in a previous study using the same RTCA assay [34]. (Summarized in Table 2). Next, to assess AFB1 toxicity to HHs with time, the viability of AFB1 treated PXB-cells was examined with WST1 assay at day 2, 6, and 14 after treatment. The results clearly indicated that a longer AFB1 treatment, especially at low concentration, produced more severe cytotoxicity (Fig 4C). For example, 2 days AFB1 treatment at 1.111 μM produced no significant cytotoxicity of PXB-cells, 6 days treatment at the same concentration significantly reduced cell viability and viability was <10% after 14 days of treatment at the same dose. These results regarding differences in severity of cytotoxicity among cell types and a time-dependent cytotoxicity strongly suggested that bioactivation of AFB1 into reactive metabolites was essential for severe cytotoxic effect of AFB1 on PXB-cells *in vitro*.

**Table 2:** Comparison of LC_50_ of AFB1 for different cell types.

Cell type	LC50 of AFB1 at day 2 (μM)
HepG2*	>75.9
HepaRG*	6.91±0.75
PHH*	1.88±0.92
PXB-cells	2.09±0.40

* Gerets 2012, PMID: 22258563 (modified).

**Fig 5:**
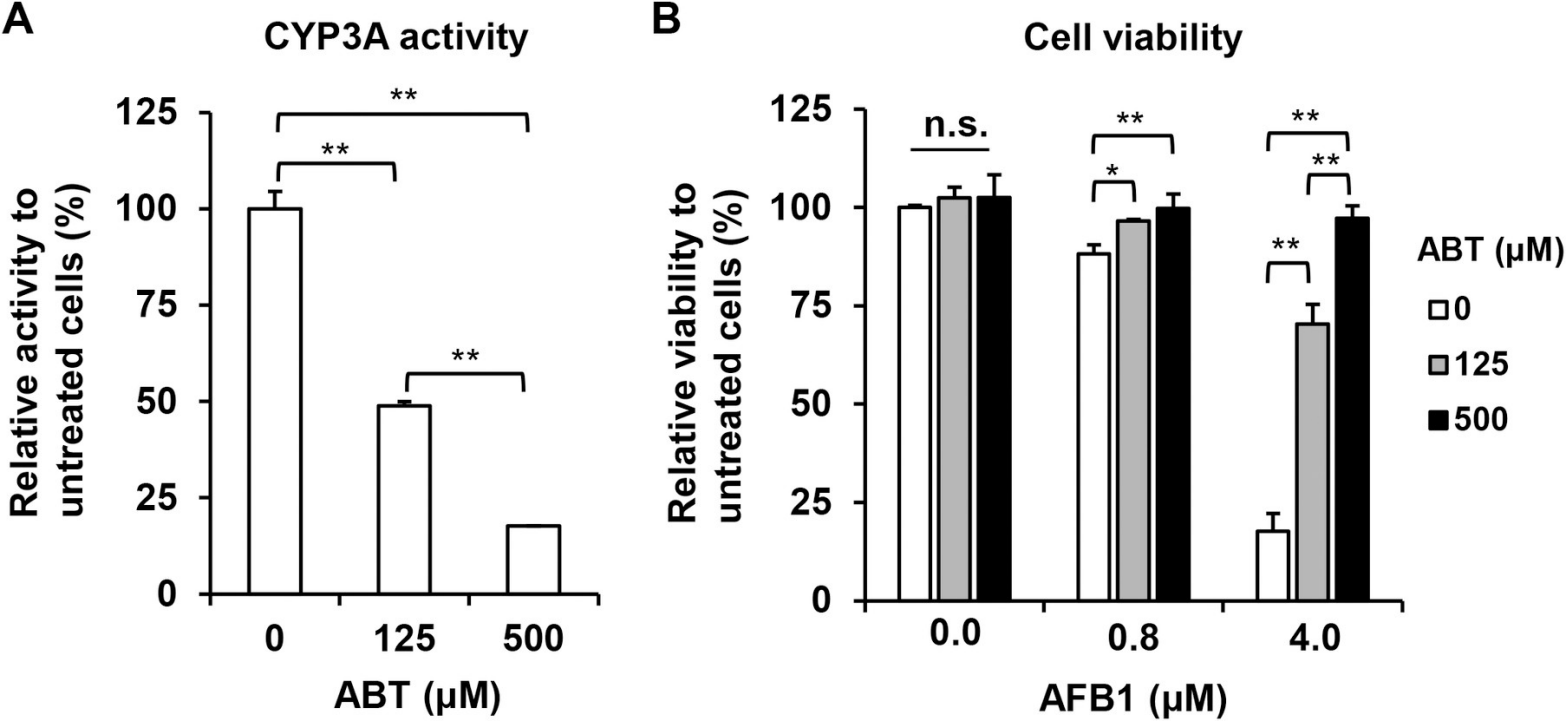
Effects of ABT on CYP3A activity and cell viability in AFB1-treated PXB-cells. (A) PXB-cells were treated with ABT at indicated dose for 5 days and CYP3A activities were measured with HPLC. (B) PXB-cells were treated with AFB1 in the presence or absence of ABT for 8 days and cell viabilities were analyzed with the WST-1 assay. Values are means ± S.D. *p <0.05, **p <0.01 (Tukey-Kramer test). n.s., not significant (*p* >0.05, Tukey-Kramer test).

**Fig 6:**
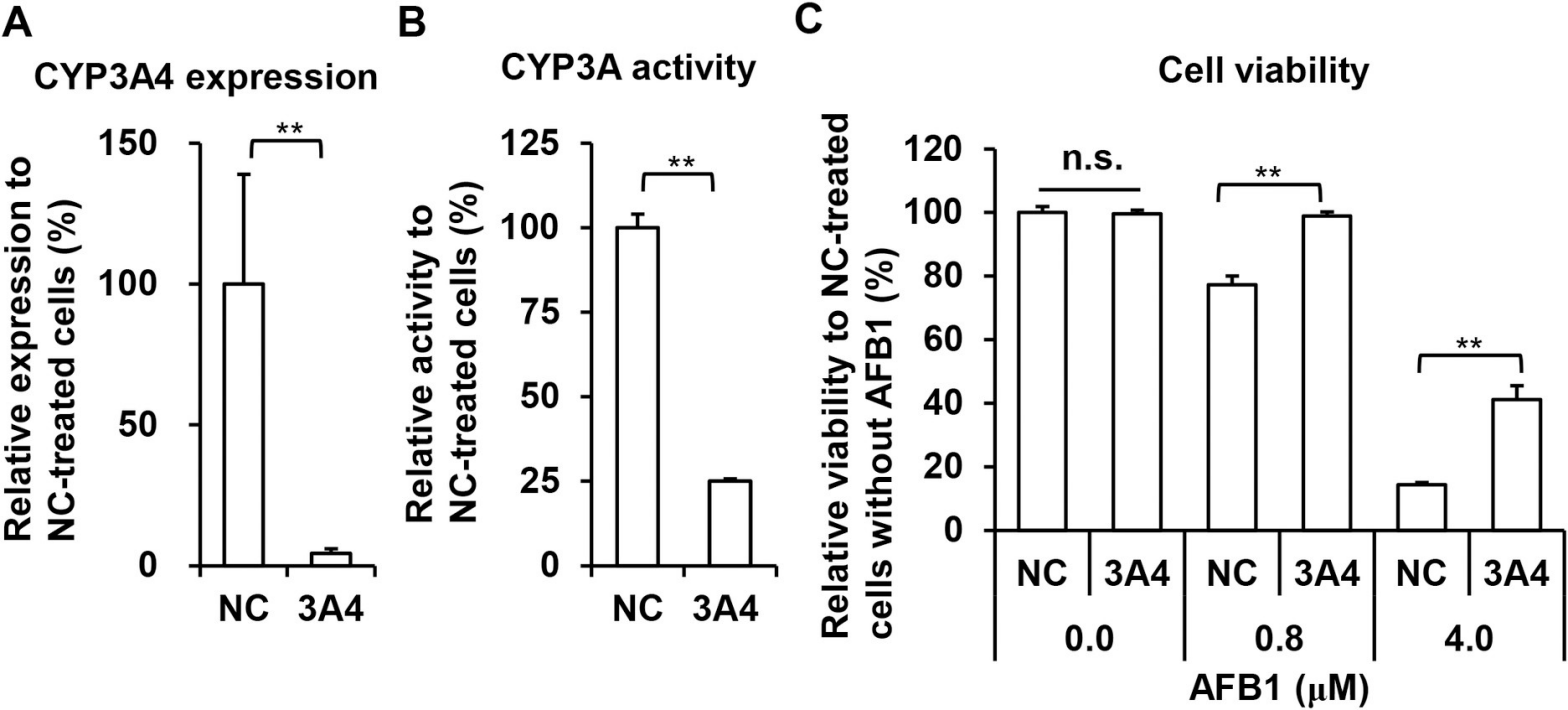
Effects of CYP3A4-specific suppression on cytotoxicity of AFB1. Expression levels of CYP3A4 mRNA (A) and CYP3A activities (B) in PXB-cells were measured by RT-qPCR and HPLC, respectively, 7 days after transfection of NC or CYP3A4-specific siRNAs (3A4). (C) Effects of CYP3A4 knock down on viabilities in PXB-cells treated with AFB1 were analyzed with the WST-1 assay. PXB-cells were treated with AFB1 at the indicated dose beginning 7 days after transfection for 6 days. Values are means ± S.D. **p<0.01 (Student’s *t* test or Welch’s *t* test). n.s.; not significant (*p*>0.05, Student’s *t* test).

To elucidate the involvement of bioactivation of AFB1 in the hepatotoxicity *in vitro*, PXB-cells were treated with AFB1 in the presence or absence of the ABT non-specific CYP inhibitor. ABT treatment at 125 and 500 μM for 5 days reduced CYP3A activity in PXB-cells to approximately 50% and 20% that of untreated cells, respectively (Fig 5A). Next, the influence of the suppression of CYP activity on AFB1-induced cytotoxicity was examined using the WST-1 assay after 8 days treatment with AFB1 in the presence or absence of ABT (Fig 5B). ABT treatment at low (125 μM) or high (500 μM) dose produced no cytotoxic effect on cultured PXB-cells. Although the cell viability was moderately or dramatically reduced by 0.8 or 4.0 μM AFB1 treatment respectively, this dose-dependent cytotoxicity was partially or completely suppressed by co-treatment with ABT at low or high dose. These results indicated that bioactivation of AFB1 mediated by CYPs was essential for AFB1 cytotoxicity at the dose levels examined in this study. Furthermore, to examine the impacts of the suppression of CYP3A4 on AFB1 cytotoxicity, PXB-cells were transfected with negative control (NC) or CYP3A4 specific siRNAs. RT-qPCR and measurement of CYP3A activity revealed that transfection of CYP3A4 specific siRNA resulted in significant reduction of CYP3A4 mRNA expression (4.3% of NC, Fig 6A) and CYP3A activity (28.0% of NC, Fig 6B) at 7 days after transfection. Finally, PXB-cells were treated with low (0.8 μM) or high (4.0 μM) dose of AFB1 for 6 days beginning 8 days after transfection of each siRNA, and cell viabilities were analyzed by WST-1 assay (Fig 6C). In the absence of AFB1, there was no difference in cell viability between NC- and CYP3A4 siRNA transfected cells. However, knock down of CYP3A4 completely inhibited moderate level cytotoxicity induced by low dose AFB1 treatment. Moreover, the severe cytotoxicity observed in PXB-cells treated with high dose of AFB1 was also partially alleviated by transfection of CYP3A4 siRNA.

## Discussion

Although the liver toxicity of AFB1 has been confirmed in many experimental animals, including rodents and nonhuman primates, adult mice are remarkably resistant to AFB1 liver toxicity [2]. mGSTA3 is constitutively expressed in liver and displays extremely high conjugating activity toward AFBO, and so is the most toxic AFB1 metabolite [35, 36]. Acute liver injury was confirmed in GSTA3 knockout mice treated with AFB1 at doses that had minimal toxic effects in wild-type mice [7]. Consistent with these previous reports, AFB1 did not affect total ALT activity levels and liver histology in the SCID mice. As with AFB1-injected SCID mice, host mouse hepatocytes in the humanized livers did not show any morphological change after AFB1 treatment. On the other hand, many vacuolated HHs and infiltrated inflammatory cells were observed in the HH area in PXB-mice treated with AFB1. These histological observations clearly suggest that species differences in sensitivity to AFB1-induced hepatotoxicity can be recapitulated in the humanized liver chimeric mice.

The serum or plasma ALT level is one of the most accepted biomarkers related to liver injury, and was previously utilized to evaluate the hepatotoxicity of test compounds on humanized liver chimeric mice [20, 21, 23]. However, conventional methods detected not only HH-derived ALTs but also host mouse cell-derived ALTs. To analyze AFB1-induced human specific hepatotoxicity in the chimeric mice with a biochemical method, we developed a novel sandwich ELISA using two different hALT1 specific mAbs. The ELISA was highly specific for hALT1, and showed no cross-reactivity to both hALT2 and mouse-derived ALTs. Consistent with histological observations, significant increase of plasma hALT1 level in AFB1-treated mice was confirmed by the novel sandwich ELISA. Such in-life phase monitoring of HH injury with a biochemical marker is very beneficial for future studies elucidating human specific hepatotoxicity of AFB1 and drug-induced liver injury with humanized liver mice.

Although the human hepatotoxicity of AFB1 have been analyzed with different established cell lines and primary HHs, significant difference in sensitivity to AFB1 induced hepatotoxicity among these *in vitro* models has been observed. One of the reasons why these cell types showed different sensitivity to AFB1 hepatotoxicity is that many CYPs, including CYP3A4, which is most responsible enzyme for bioactivation of AFB1, are expressed in the established cell lines at lower levels comparing with primary HHs. A previous study showed that cells with higher CYP activities showed lower LC_50_ value of AFB1 [34]. It has been demonstrated that the CYP3A activity in cultured PXB-cells is comparable to that in original primary HHs used for xenotransplantation [27, 29]. Reflecting the stable expression of CYP3A4 in PXB-cells, the present results revealed PXB-cells exhibited highly comparable sensitivity to primary HHs regarding the previously reported AFB1-induced hepatotoxicity [34]. Moreover, the results of ABT treatment demonstrated that CYP-mediated bioactivation of AFB1 was essential for AFB1 cytotoxicity to PXB-cells *in vitro* at dose levels examined in this study. As with ABT treatment, CYP3A4 specific suppression by siRNA resulted in reduced hepatotoxicity of AFB1 to PXB-cells. The findings indicate that CYP3A4 is a dominant enzyme for the bioactivation of AFB1 in PXB-cells. Interestingly, the siRNA showed more inhibitory effect on CYP3A activity than low dose ABT treatment. However, PXB-cells treated with ABT were more resistant to AFB1-induced hepatotoxicity than the cells transfected with the siRNA. The difference in the specificity for CYP inhibition between ABT treatment and siRNA transfection seemingly bolsters the previous suggested contribution of the other CYPs to the production of reactive metabolites of AFB1 [3].

In general, it is difficult to detect the hepatotoxicity of test compounds, which require bioactivation by metabolic enzymes with a long-term exposure at a low dose, with lack of toxicity in short-term treatment. Even though primary HHs are considered a gold standard for *in vitro* toxicity study, many hepatocyte characteristics, including CYP activity, quickly diminish *in vitro* [37]. However, in terms of CYP3A activity, PXB-cells exhibited comparable CYP3A activity to freshly isolated or cryopreserved primary HHs, at least until 3 weeks after plating, as previously reported [27]. The present results clearly demonstrated that 2 or 6 days exposure to 0.37 μM AFB1 did not show any effect on PXB-cells, but that 14 days treatment at the same dose reduced cell viability to <40%. These findings suggest that PXB-cells are a valuable tool to validate the time-dependent cytotoxic effect of relatively low levels of AFB1 on HHs.

In summary, we provide the first demonstration that humanized liver chimeric mice are useful for the detection of HH specific cytotoxicity of AFB1 using histological examination and biochemical analysis with a novel ELISA specific for hALT1. The data also indicate that fresh HHs isolated from the chimeric mice exhibit comparable sensitivity to primary HHs regarding AFB1-induced cytotoxicity depending on bioactivation by CYPs, and that the fresh HHs are useful for the detection of a cytotoxic effect of long-term exposure to a low dose of AFB1. PXB-mice and derived HHs appear to be a more reliable *in vivo* and *vitro* platform for the study of AFB1 hepatotoxicity to HHs.

## Supporting information

S1 File (DOCX)
