# Supplementary material for: Detection of acute toxicity of aflatoxin B1 to human hepatocytes in vitro and in vivo using chimeric mice with humanized livers
Source: PLoS One. 2020 Sep 23;15(9):e0239540. doi: 10.1371/journal.pone.0239540 (PMC7510964; doi:10.1371/journal.pone.0239540)
Supplement: S1 File — (DOCX) [file pone.0239540.s001.docx]

**S1 Materials and Methods:**

**Preparation of ALT sample**

Blood samples were collected from the SCID mice that had been injected with CCl_4_ (50 mg/mL) or a hydrodynamic injection (HDI; saline, 10% of body weight). Serum was prepared (CCl_4_-SCID serum and HDI-SCID serum). Human sera from chronic liver disease patients with or without human albumin depletion treatment were used as samples containing hALTs (human serum and albumin-negative [Alb (-)] human serum). Serum was also prepared from a PXB-mouse after hydrodynamic injection (15% body weight) of saline (HDI-PXB serum). These sera were used to confirm reactivity of our hALT1 specific ELISA as described below. The ALT activity of each serum sample was measured using FUJIFILM DRI-CHEM7000 (Fujifilm). The concentration of hALT1 in Alb (-) human serum was quantified by mass spectrometry. The serum protein was alkylated and digested with trypsin as previously described [1], and 2.5 pmol of the stable-isotope labeled peptide FAFEER* (with the asterisk indicating the ^13^C- and ^15^N-isotopic-labeled amino acid) was spiked as the internal reference into tryptic digest derived from 30 uL of serum. The tryptic digests were analyzed using a nano-liquid chromatography pump (AMR) connected to a triple quadrupole mass spectrometer (4000QTRAP, AB Sciex). The mass spectrometer was operated in multiple reaction monitoring mode with eight transitions for FAFEER (y2 to y5) derived from both endogenous and isotope-labeled peptides. The amounts of hALT1 in the serum were quantified as 84.3±10.7 fmol/uL by calculating the ratios of the peak areas to those of the isotope-labeled peptide. The serum quantified by mass spectrometry was used as a standard sample (gold standard of hALT1) for the following sandwich ELISA.

**Establishment of mouse anti-human ALT1 monoclonal antibodies (anti-hALT1 mAbs)**

Recombinant hALT1 and 2 bearing C-terminal 6×Histidine tag (rhALT1 and rhALT2) were prepared with a baculovirus-based expression system and rhALT1 was used as an immunogen to establish anti-hALT1 mAbs. The chemically synthesized DNA fragments for hALT1 (Genbank: NM_005309.3) and hALT2 (Genbank: NM_133443.4) with C-terminal 6×Histidine tag were obtained from GenScript Japan K.K. They were cloned into a pFastBac-1 baculovirus expression vector. The bacmid containing the hALT1 or hALT2 bearing 6×Histidine tag sequence was generated using the pFastBac plasmid containing hALT1 or hALT2 by carefully following the manufacturer’s instructions (“Bac-to-Bac basculovirus expression system”; Thermo Fisher Scientific K.K.).

Sf9 insect cells (6×10^6^) cultured in a 100 mm-tissue culture plate were transfected with 100 μg of the hALT1/bacmid or hALT2/bacmid. After 48 h incubation, the culture supernatant containing the first generation of baculovirus bearing hALTs with the 6×Histidine tag (hALT1 baculovirus or hALT2 baculovirus) was collected. The baculovirus was amplified by secondary infection to a 100 mL suspension culture of 5×10^7^ Sf9 cells to obtain the virus titer of nearly 1×10^8^ plaque forming units (pfu)/mL. Sf9 cells cultured in suspension at a density of 2×10^6^ cells/mL were infected with the hALT1 or hALT2 baculovirus using a multiplicity of infection of 10. After a 72-h incubation, rhALT1 and rhALT2 generated in the Sf9 cells were respectively extracted and purified by column chromatography using Ni-NTA agarose resin (Qiagen K.K.-Japan).

After immunization of mice with the purified rhALT1, spleen cells were isolated and fused with mouse myeloma NS-1 cells using conventional methods. Primary hybridomas were selected by direct ELISA against the purified rhALT1 and rhALT2. Immunoblots against the purified [2] rhALT1, rhALT2 or CCl_4_-SCID serum (mouse ALT1/2) were performed for the positive wells to confirm their specificity. Hybridomas that specifically reacted with rhALT1 were chosen, followed by cloning with limited dilution to establish the monoclonal hybridoma producing anti-hALT1 mAbs.

**References**

1. Kamiie J, Ohtsuki S, Iwase R, Ohmine K, Katsukura Y, Yanai K, et al. Quantitative atlas of membrane transporter proteins: development and application of a highly sensitive simultaneous LC/MS/MS method combined with novel in-silico peptide selection criteria. Pharmaceutical research. 2008;25(6):1469-83. <https://doi.org/10.1007/s11095-008-9532-4> PMID: 18219561.

2. Kohler G, Milstein C. Continuous cultures of fused cells secreting antibody of predefined specificity. Nature. 1975;256(5517):495-7. <https://doi.org/10.1038/256495a0> PMID: 1172191.
